# Robotic repair of off-midline abdominal wall hernias: a single institution consecutive case series

**DOI:** 10.1007/s10029-025-03476-8

**Published:** 2025-09-29

**Authors:** Priti Dutta, Gina Kim, Nathan English, Tapasya Katta, Gurudatta Naik, Margaux Mustian, Britney Corey, Abhishek D. Parmar

**Affiliations:** Division of Gastrointestinal Surgery, Department of Surgery, University of Alabama at Birmingham, 1808 7th Avenue South Boshell Diabetes Building #525, 35233 205.934.7315 Birmingham, AL United States

**Keywords:** Off-midline hernia, Flank hernia, Robotic, Extraperitoneal repair, Outcomes

## Abstract

**Introduction:**

While off-midline hernias represent only a small percentage of abdominal wall defects, symptoms can be debilitating for the affected patients, and repair portends substantial operative challenges for the surgeon. There are no large-scale case series that describe outcomes with extraperitoneal repair using the robotic approach. The objective of this study was to describe our experience of patients undergoing robotic repair of off-midline hernias at a tertiary care medical center.

**Methods:**

This study was a retrospective review of patients who underwent elective robotic repair of off-midline hernias from June 2019 to October 2024. All adults (≥ 18 years old) diagnosed with a primary (no prior repair) or recurrent off-midline hernia were included. Patient demographics, preoperative clinical variables (smoking status, BMI, ASA score, co-morbidities, presence of pain, hernia type, history of prior hernia repair, type of prior flank operation, and dimensions of the hernia on CT scan), operative variables (perioperative regional pain blocks, operative approach, type and dimensions of mesh used), and postoperative outcomes (hospital length of stay (LOS), follow-up duration, hernia recurrences, and complications including wound occurrences and chronic pain defined as pain > 3 months postop) were abstracted from a prospectively maintained hernia database. Univariate analyses were used to measure and describe all covariates and outcomes.

**Results:**

There were 43 patients included in the study. Patients had an average age of 57.5 years and an average BMI of 32.8 kg/m2. 81% of the cohort identified as White race and 65% were female. 26% of the cohort had diabetes mellitus (average HbA1c 5.8), 7% had a smoking history, and 9% had COPD. Most hernias were incisional (51%) or traumatic (47%). Average hernia length and width were 8.0 ± 4.5 and 6.7 ± 2.7 cm. 98% had a clean wound classification. Fascial closure was performed in 86% of the cases and a mesh was placed in 98% of the cases. Average mesh length and width were 21.2 ± 5.9 and 21.1 ± 5.1 cm. There were 6 (14%) hybrid procedures, where the hernia was repaired via a combination of a robotic and open approach. Average hospital length of stay was 1.9 days and average follow up was 4.4 months. There were two (5%) recurrences. There were 15 (35%) postoperative complications. The most common complications were seroma (14%), hematoma (7%) and persistent pain > 3 months (5%). One patient (2%) developed an abscess, two patients (5%) had a nerve injury, and one patient (2%) had a postoperative small bowel obstruction.

**Conclusion:**

Robotic off-midline hernia repair can be performed with minimal morbidity. Most common complication was postoperative seroma. Surgical outcomes were similar to existing literature on outcomes following open repair. Surgeons performing this repair should appropriately counsel their patients on the risks of pain and recurrence postoperatively.

## Introduction

Off-midline hernias are a rare subset of abdominal wall defects that represent a unique set of operative challenges due to their location and proximity to anatomical structures [1]. According to the European Hernia Society (EHS) classification for primary and incisional abdominal wall hernias established in 2009, off-midline hernias are defined as hernias occurring lateral to the rectus sheath [2]. They present unique anatomical challenges in repair due to the proximity of bony prominences and vital retroperitoneal and pelvic neurovascular structures [3, 4]. They most commonly arise as incisional hernias after urologic/other flank surgeries [5, 6], but may also present because of trauma [7–13] or congenital abdominal wall musculature abnormalities [14–16]. Risk factors for the development of incisional off-midline hernias include high BMI, use of self-retaining retractor, inability to preserve neurovascular bundles, en masse wound closure, surgical site infections, and postoperative abdominal distension [17].

Due to the rarity of off-midline hernias and the complexity of repair, there is no consensus on the optimal approach for repair [1, 4]. Various repair techniques have been described, including open [8, 9, 18–20], minimally invasive [12, 13, 21], and hybrid approaches [13, 22, 23]. The largest consecutive series on flank hernia repair to date by Salvino et al. has provided extensive insight into the outcomes with an open extraperitoneal approach to repair. While that study is particularly valuable, there are no other larger scale case series that describe outcomes with extraperitoneal repair using the robotic approach.

Given the expansive popularity of the robotic approach for a variety of hernia repairs [8, 24], we aimed to explore the outcomes of this modality for off-midline hernia repair. Our study’s goals are to describe our experience with robotic off-midline hernia repair. First, we describe the technical nuances of the robotic approach which slightly differs from the open approach. In addition, we describe our clinical outcomes with repair.

## Methods

### Study design

This was a retrospective single institution case series analysis of prospectively collected data on patients who underwent robotic off-midline hernia repair. Patient data was maintained in the Abdominal Core Health Quality Collaborative (ACHQC) database. Relevant attributes such as demographics, comorbidities, perioperative complications, and recurrence were then extracted from the database. The Institutional Review Board at the University of Alabama at Birmingham approved this study (#300003313).

### Patient population

We performed a retrospective single-surgeon case series of patients > 18 years who underwent robotic off-midline hernia repair from June 2019 to October 2024 at the University of Alabama at Birmingham (UAB). UAB is a tertiary referral center that serves a wide catchment area that includes Alabama, Florida, Georgia, Tennessee, and Mississippi. Patient cases and characteristics were identified through a prospectively maintained database on the Abdominal Core Health Quality Collaborative (ACHQC). For the purposes of this analysis, off-midline hernias were defined as any isolated off-midline hernias including subcostal, flank, and iliac hernias, also defined by the EHS classification zone as L1, 2, and L3 respectively. Data on patient demographics, comorbidities, and hernia characteristics were collected. Any data missing from the ACHQC database were supplemented with individual chart review. CT measurements were obtained from radiology read or operative note; where none available, hernia length and width measurements were obtained manually. Follow-up was defined as the last patient visit or last available postoperative CT scan.

### Preoperative optimization

Patients were evaluated in clinic by the attending surgeon and a thorough history and physical exam in conjunction with cross sectional imaging were used to confirm the diagnosis of an off-midline hernia. Patients were counseled on smoking cessation, diabetes control, and weight loss as appropriate for preoperative optimization. Patients also underwent preoperative medical evaluation as needed based on their comorbidity profile and level of hernia complexity.

### Operative approach

All patients were enrolled in our enhanced recovery protocol, which includes 14 perioperative components and has previously been shown to reduce length of stay, reduce narcotic use, and lead to fewer complications [25]. In the preoperative area all patients were offered an ultrasound guided regional block by the anesthesia team with the option to opt in or out based on patient preference. All patients received appropriate preoperative antibiotics and deep vein thrombosis prophylaxis to include 5,000 units of subcutaneous heparin and sequential compression devices.

**Fig. 1:**
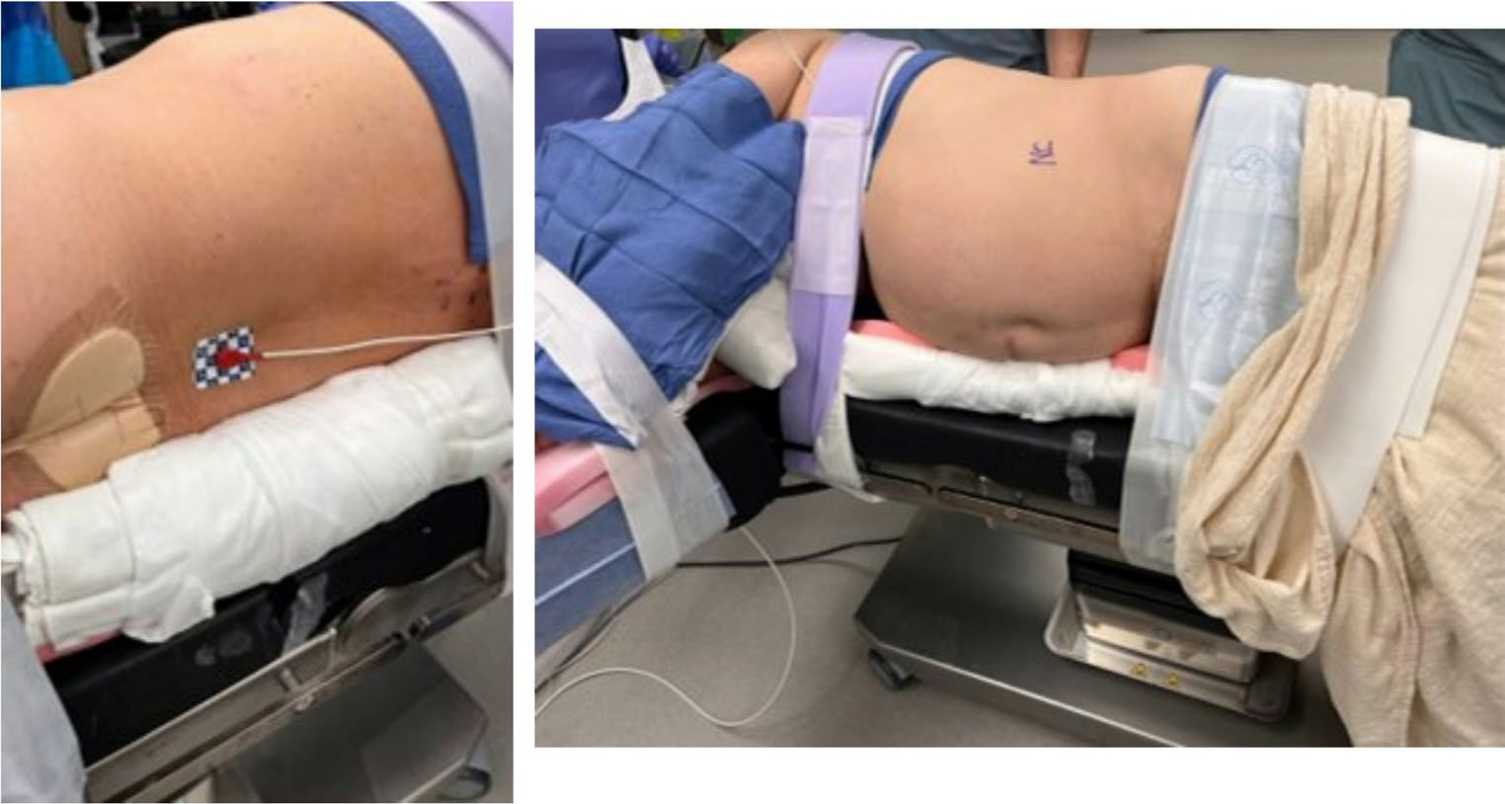
Operative positioning

**Fig. 2:**
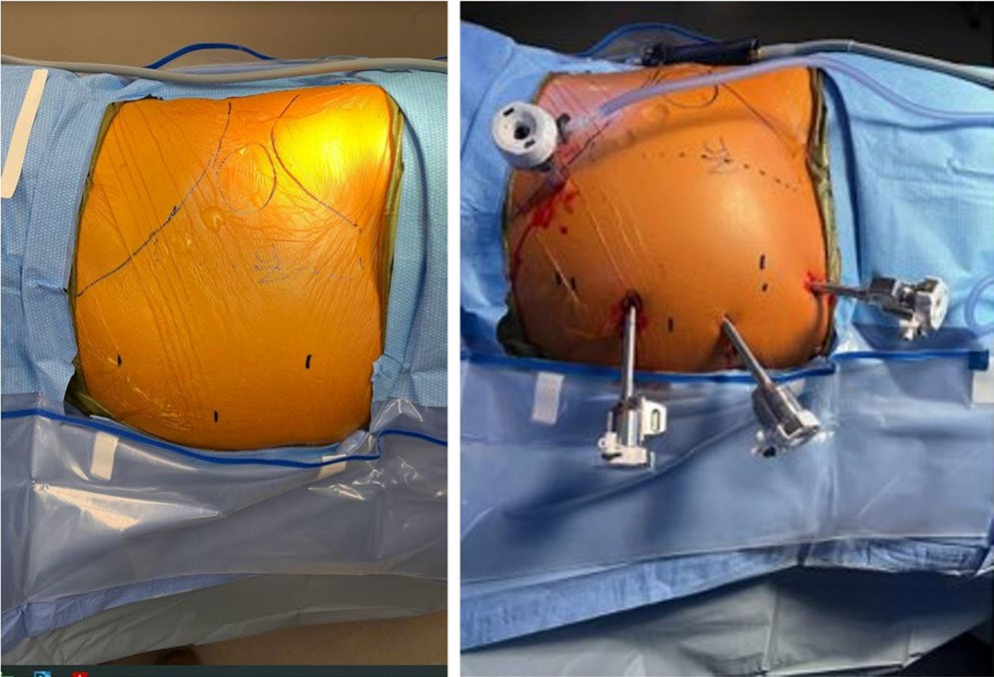
Port placement

**Fig. 3:**
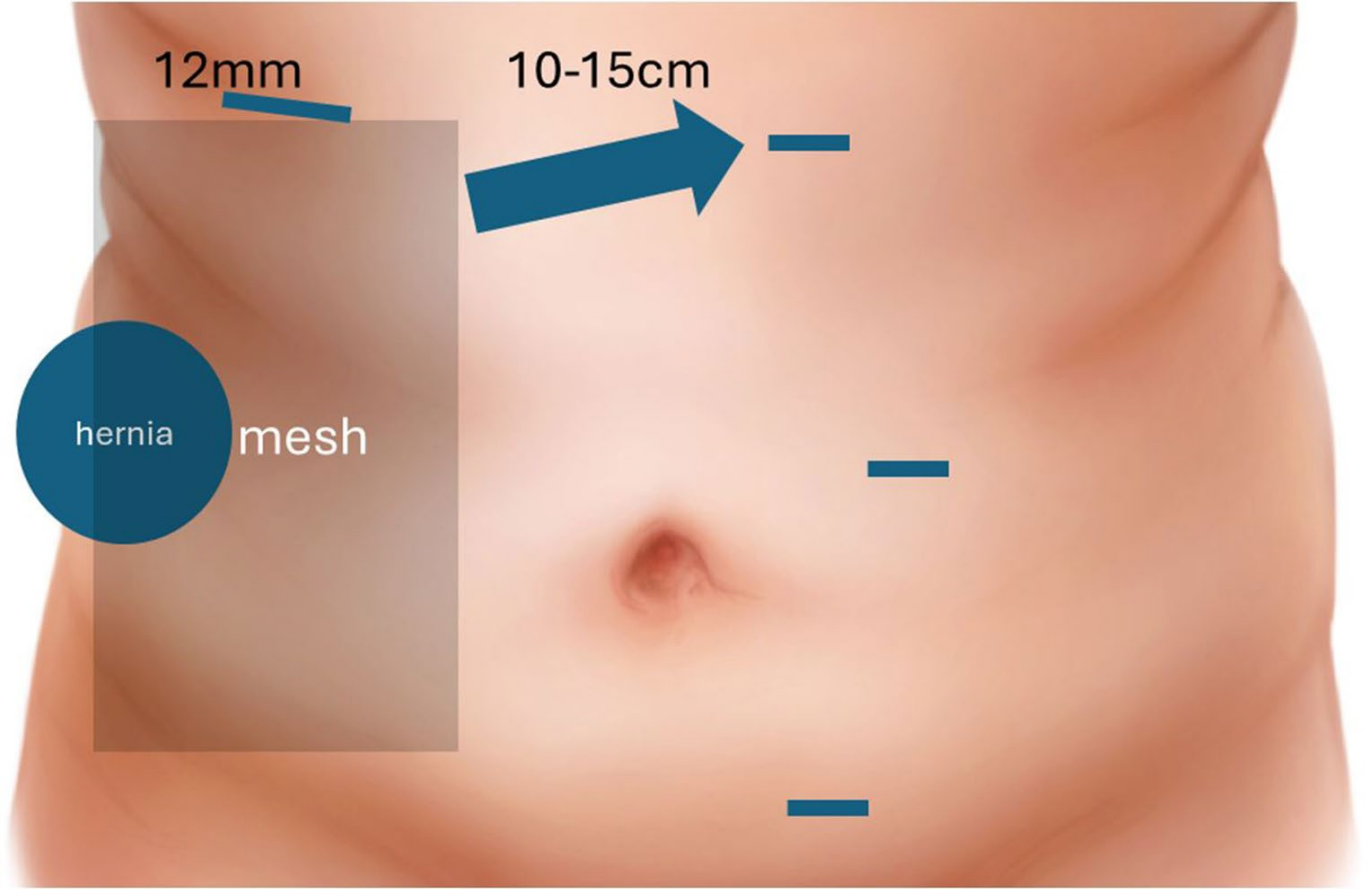
Suggested port placement

Patient positioning for this operation is critical and nuanced, as demonstrated in Figs. 1, 2 and 3. In the operating room, patients were placed in the off-midline decubitus position with an axillary roll. Notably, it is our preference to not utilize a bean bag as it can limit robotic arm range of motion due to external collisions with the bean bag. In lieu of this, we prefer rolled up towels or sheets to be placed along the patient’s anterior abdomen and back and tucked into the patient’s drawsheet to stabilize the patient in the off-midline decubitus position. Patient is then secured to the table using standard straps. In addition, a pink pad is utilized to further ensure patient stability. Both arms are then extended; the patient’s lower arm is supported on a standard padded arm rest while their overlying arm is placed on supportive pillows and secured in place with tape. Again, this is done to avoid the possibility for instrument arm clashes that can occur with a larger, higher profile supportive structure like a Mayo stand. Finally, we avoid flexing the bed as this can make robotic suturing more challenging, particularly for larger defects that will be under increased tension.

A transabdominal preperitoneal (TAPP) approach was used to repair the hernias. Abdominal access is obtained with a Veress needle first and only after this insufflation is performed do we plan for port placement. This is critical as the abdominal wall contour changes with insufflation and additional space is obtained beneath the costal margin and above the iliac crest for port placement. One we have achieved maximal insufflation, we mark key landmarks. First we outline the margins of the hernia, then a medial and off-midline margin of 10 cm to accommodate for mesh overlap, and finally, the iliac crest and costal margin. We use these boundaries to plan port placement and to avoid external collisions with the patient’s bony prominences. A 5 mm optical viewing trocar is advanced at the costal margin and then three transabdominal 8 mm robotic trocars are then placed under direct visualization. The 5 mm port is valuable not only to gauge optimal port placement but also as it will be upsized to a 12 mm port for later heavyweight mesh placement (Figs. 2 and 3). The table is airplaned with hernia side up as needed to facilitate exposure. A peritoneal flap was raised to ensure at least 10 cm overlap circumferentially to access the preperitoneal space. The hernia defect was closed using 0 absorbable self-locking suture in a running fashion. An appropriately sized, generally heavyweight microporous polypropylene mesh was placed in the preperitoneal pocket and the peritoneum was closed using a running 2 − 0 absorbable self-locking suture. In select cases where the surgeon deemed that excessive tension on large defects was present, a hybrid approach was taken where an open incision was made and a sandwich repair performed.

### Statistical analysis

Standard statistical methods and descriptive statistics were used for this study. For continuous variables, mean values were reported with corresponding standard deviations. For categorical variables, percentages were reported. All statistical analyses were performed using SAS 9.4 (SAS Institute, Cary, NC).

## Results

### Patient demographics

A total of 43 robotic off-midline hernia repairs were performed by two surgeons during the research period. Baseline demographics and patient characteristics are outlined in Table 1. Patients had an average age of 57.5 years and an average BMI of 32.8 kg/m². 65% of the cohort were female. Some patients had significant comorbidities, such as smoking history (6.98%), diabetes mellitus (25.58%, average HbA1c of 5.80 ± 1.03), and COPD (9.3%). There were 35 (81%) patients who identified as White race. The most common insurance type was Medicare (42%) followed by Blue Cross Blue Shield (35%). Most patients (93%) were ASA class II or III.

**Table 1:** Patient demographics

	*n* = 43
Age (Years)	57.51 ± 13.41
Gender (Female)	28 (65.1%)
BMI (kg/m²)	32.78 ± 5.13
Smoking History	3 (6.98%)
Diabetes	11 (25.58%)
HbA1c	5.80 ± 1.03
COPD	4 (9.3%)
Race*	
White	35 (81.4%)
Native Hawaiian	1 (2.33%)
Black	7 (16.28%)
Employment	
Employed	12 (27.91%)
Unemployed	4 (9.3%)
Disabled	10 (23.26%)
Retired	12 (27.91%)
Unknown	5 (11.63%)
Insurance	
Medicare	18 (41.86%)
Medicaid	4 (9.30%)
Blue Cross Blue Shield	15 (34.88%)
Self pay	1 (2.33%)
Other	5 (11.64%)
ASA Classification	
I	1 (2.33%)
II	11 (25.58%)
III	29 (67.44%)
IV	2 (4.65%)

*BMI * body mass index, *HbA1c* hemoglobin A1C, *COPD* chronic obstructive pulmonary disease, *ASA* american society of anesthesiologists *Race was self-reported

### Operative characteristics

The operative characteristics are listed in Table 2. Most hernias were incisional (51%) or traumatic (47%). There were 30 patients (70%) who had a prior flank operation. These operations were most commonly orthopedic (33%) or urologic (9%). Preoperative pain was present in 63% of patients. All but one case (98%) had a clean wound classification. A drain was placed in 5 (12%) patients. A majority of the patients (53%) were diagnosed with EHS class L4 (lumbar) hernias. Representative images are shown in Table 3.

**Table 2:** Operative characteristics

	*n* = 43
Preoperative Pain Present	27 (62.79%)
Type of Hernia	
Incisional	22 (51.16%)
Traumatic	20 (46.51%)
Spontaneous	1 (2.33%)
Hernia defect length (cm)	7.99 ± 4.46
Hernia defect width (cm)	6.70 ± 2.67
EHS Classification	
L1 (subcostal)	0 (0.00%)
L2 (flank)	4 (9.30%)
L3 (iliac)	1 (2.33%)
L4 (lumbar)	23 (53.49%)
L1-4	1 (2.33%)
L2-4	6 (1.40%)
L3-4	8 (1.86%)
Prior Hernia Repair	13 (30.23%)
Prior Flank Operation	30 (69.77%)
Type of Prior Operation	
Orthopedic	14 (32.56%)
Urologic	4 (9.30%)
Other	12 (27.91%)
N/A	13 (30.23%)
Wound Classification of Clean	42 (97.67%)
Mesh Type	
Bard Heavyweight Mesh (Polypropylene large pore heavyweight)	31 (72.09%)
Bard Soft Mesh (Polypropylene macroporous lightweight)	2 (4.65%)
Bard Mesh (Polypropylene medium pore heavyweight)	3 (6.98%)
Parietex Heavyweight Mesh (Polyester macroporous heavyweight)	4 (9.30%)
Progrip (Polyester large pore midweight)	2 (4.65%)
No Mesh Used	1 (2.33%)
Mesh Length/Width (cm)	21.21 ± 5.89/21.14 ± 5.12
Fixation Used	7 (16.28%)
Fascial Closure	37 (86.05%)
Drain used	5 (11.63%)
Preoperative Pain Block	
Unioff-midline Erector Spinae	1 (2.33%)
Bioff-midline Erector Spinae	6 (13.95%)
Unioff-midline Quadratus Lumborum	2 (4.65%)
Bioff-midline Quadratus Lumborum	20 (46.51%)
Intrathecal	2 (4.65%)
Paravertebral (T8-T10)	1 (2.33%)
None	11 (25.58%)
Hybrid Procedure Done	6 (13.95%)

**Table 3:** Postoperative outcomes

	*n* = 43
Length of Stay (days) Time to last follow up (months)	1.90 ± 2.19 4.40 ± 8.64
Hernia Recurrence	2 (4.65%)
Complications	15 (34.88%)
Seroma	6 (13.95%)
Hematoma	3 (6.97%)
Abscess	1 (2.33%)
Persistent Pain	2 (4.65%)
Nerve Injury*	2 (4.65%)
Small Bowel Obstruction	1 (2.33%)
Postoperative Pain	10 23.26%)

*Nerve injury was defined as pain that was diagnosed by the operating surgeon to be neuropathic in nature (burning) and that was not present prior to the operation. Unremitting pain was referred to interventional radiology for cryoablation of the affected nerve

A majority of the patients (74%) received a preoperative pain block. A fascial closure was performed in 86% of the cases. A mesh was placed in 98% of the cases. The most commonly used mesh was a heavyweight polypropyelene mesh (72%). The average hernia defect length and width were 8.0 ± 4.5 and 6.7 ± 2.7 cm. The average mesh length and width were 21.2 ± 5.9 and 21.1 ± 5.1 cm. Mesh fixation was performed in 7 (16%) cases. Of note, there were 6 (14%) hybrid procedures, where the hernia was repaired via a combination of robotic and open approach.

### Postoperative outcomes

Postoperative outcomes are summarized in Table 4. The average length of stay was 1.9 days. Patients were followed for an average of 4.4 months. At the time of last follow up, there were two (5%) hernia recurrences. There were 15 (35%) cases with postoperative complications. The most common complication was a seroma, of which 6 out of the 15 patients that had postoperative complications experienced. Three patients (7%) experienced hematomas, two (5%) noted persistent pain that lasted beyond 3 months, two patients experienced nerve injuries, one suffered from an abscess at the surgical site, and one experienced a postoperative small bowel obstruction. Of all patients, 23.3% experienced postoperative pain, which was defined as pain lasting more than 3 months after the operation.

**Table 4:**
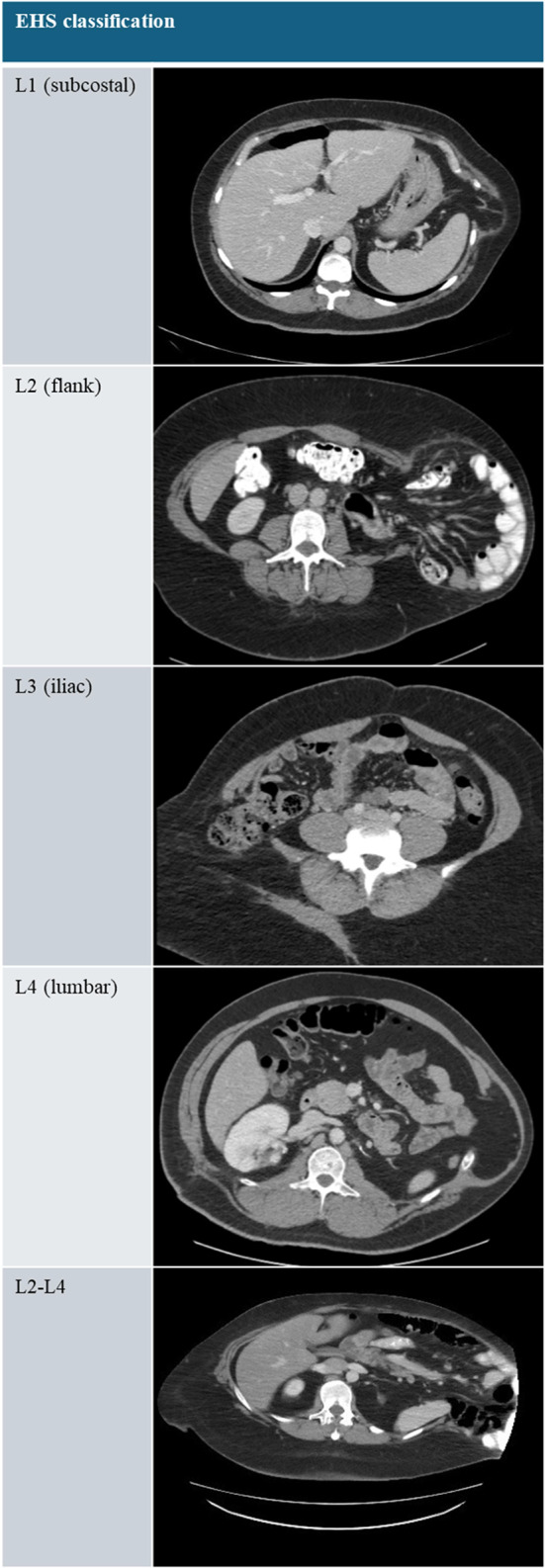
Representative CT images of European Hernia Society classification system for abdominal wall hernias

## Discussion

To our knowledge, this study represents the largest published consecutive case series of robotic off-midline hernia repairs. This study demonstrates minimal morbidity with the robotic approach and the feasibility of reasonable outcomes even for larger defects. In this series, off-midline hernias were most commonly due to prior surgical incisions or traumatic injuries. Most cases were able to be performed entirely robotically, while a small minority of the cases did require a hybrid approach. Primary fascial closure was achieved in 86% of the cases and a mesh was used in all but one of the cases. The most common postoperative complications were surgical site occurrence and persistent postoperative pain. However, there was a high rate of preoperative pain (63%) associated with the hernia as well. There was a very low hernia recurrence rate (< 5%) observed during the follow up period, in keeping with published reports with the open approach. Of note, patients with denervation bulging secondary to neurogenic amyotrophy were diagnosed with physical exam or with confirmatory provocative hernia ultrasound in cases where the diagnosis was unclear. These patients were excluded from our cohort and only those with true fascial defects were included.

Previous studies have demonstrated that minimally invasive approach to off-midline hernia repair is safe and feasible [12, 21]. However, these prior studies have been limited by dated technical approaches such as intraperitoneal underlay mesh placement and the lack of hernia defect closure. In the past few years, the feasibility of the robotic approach has been described but in very small case series [26, 27]. This study builds upon this foundation by not only providing a larger cohort but also by introducing a modern extraperitoneal technique facilitated by robotic technology. The superiority of the extraperitoneal compared to the intraperitoneal approach can certainly be called into question, but several studies from midline ventral hernia repair support this approach [28–30]. For off-midline abdominal wall hernias, we believe that the extraperitoneal approach is particularly valuable as it can minimize the need for mesh fixation in a high-risk operative field where nerves may be vulnerable. In addition, this is the exact approach performed in the largest open series to date [20], which was performed with excellent clinical outcomes. Nonetheless, additional research with longer term follow-up is needed to confirm similar benefits specific to the robotic approach to off-midline hernia repairs.

In fact, findings in the current study mirror those of the largest series of open extraperitoneal flank hernia repairs reported by Salvino et al. [20]. In their review of 142 patients with flank or lumbar hernias undergoing an open extraperitoneal repair, they reported a hernia recurrence rate of 3.5% at an average of 30 month follow up. The most common postoperative morbidity was chronic pain, with 21.2% of the patients experiencing pain more than 6 months after the surgery. Our robotic experience resulted in a similarly low hernia recurrence rate (4.7% vs. 3.5%) and a high rate of chronic postoperative pain (23.2% vs. 21.2%), with the caveat that our follow up was much shorter compared to Salvino et al. However, we also observed improvements in the rates of length of stay (1.9 vs. 5.5 days) as well as wound infection (2.3% vs. 8.3%) in the current study [20]. These findings are well-documented global benefits of the minimally invasive approach when compared to the open approach, so it is not surprising that our cohort experienced these benefits. The similar complication profile of recurrence and chronic pain are reassuring, overall suggesting that, with these complications being equal, perhaps the robotic extraperitoneal flank hernia repairs may be superior to the traditional open approach where feasible.

Finally, our study adds to the existing literature by providing some technical insight into the performance of robotic off-midline hernia repairs. For example, conventional strategies for positioning such as the use of a bulky bean bag for off-midline decubitus positioning or extreme flexing of the bed should be abandoned. Several positional and nuances in port placement are also mandatory to understand to avoid external collisions with the robotic arms. Furthermore, we believe that widely accessing the extraperitoneal space robotically can safely eliminate the need to fixate the mesh using bone anchors, which have the potential for extreme morbidity when performed improperly.

There are several limitations inherent to this study. This was a single institution study and the findings may not be immediately generalizable. There are inherent biases present in this retrospective study—however, this was mitigated to the best possible extent by including all consecutive robotic off-midline hernia repairs performed during the study period. This was a case series and as such there was no direct comparison to other approaches such as open or laparoscopic techniques. The follow-up interval was relatively short, which may partly explain the low rate of hernia recurrence. Our institution is a quaternary referral center for complex hernia, and most of our patients have to travel a significant distance for follow-up. We do not perform routine follow-up longitudinally but do advise patients to self-monitor for recurrent symptoms, as strategy previously validated [31]. Additionally, there is a lack of patient reported outcomes regarding quality-of-life following hernia repairs, which for this operation, is particularly valuable given the persistence of chronic pain. Regardless, this study is the first of its kind examining outcomes of robotic off-midline hernia repairs, and confirms robotic surgery as a novel, safe, and feasible option.

## Conclusion

Robotic surgery may provide a safe and feasible novel approach to the challenging task of repairing off-midline hernias. Robotic off-midline hernia repair can be performed with minimal morbidity with outcomes similar to the outcomes of open repairs that have previously been reported in the literature. Surgeons performing this repair should appropriately counsel their patients on potential complications including postoperative seromas, chronic pain, and recurrence.
